# Normalized Clinical Severity Scores Reveal a Correlation between X Chromosome Inactivation and Disease Severity in Rett Syndrome

**DOI:** 10.3390/genes15050594

**Published:** 2024-05-08

**Authors:** Jonathan K. Merritt, Xiaolan Fang, Raymond C. Caylor, Steven A. Skinner, Michael J. Friez, Alan K. Percy, Jeffrey L. Neul

**Affiliations:** 1 Department of Pediatrics, Vanderbilt University Medical Center, Nashville, TN 37232, USA; jonathan.merritt@vumc.org; 2 Department of Pathology, Henry Ford Health System, Detroit, MI 48202, USA; xfang1@hfhs.org; 3 Greenwood Genetic Center, Greenwood, SC 29646, USA; rcaylor@ggc.org (R.C.C.); sas@ggc.org (S.A.S.); friez@ggc.org (M.J.F.); 4 Department of Pediatrics, University of Alabama at Birmingham, Birmingham, AL 35294, USA; apercy@uabmc.edu; 5 Vanderbilt Kennedy Center, Vanderbilt University Medical Center, Nashville, TN 37232, USA

**Keywords:** Rett syndrome, X chromosome inactivation, longitudinal modeling

## Abstract

Rett Syndrome (RTT) is a severe neurodevelopmental disorder predominately diagnosed in females and primarily caused by pathogenic variants in the X-linked gene *Methyl-CpG Binding Protein 2* (*MECP2*). Most often, the disease causing the *MECP2* allele resides on the paternal X chromosome while a healthy copy is maintained on the maternal X chromosome with inactivation (XCI), resulting in mosaic expression of one allele in each cell. Preferential inactivation of the paternal X chromosome is theorized to result in reduced disease severity; however, establishing such a correlation is complicated by known *MECP2* genotype effects and an age-dependent increase in severity. To mitigate these confounding factors, we developed an age- and genotype-normalized measure of RTT severity by modeling longitudinal data collected in the US Rett Syndrome Natural History Study. This model accurately reflected individual increase in severity with age and preserved group-level genotype specific differences in severity, allowing for the creation of a normalized clinical severity score. Applying this normalized score to a RTT XCI dataset revealed that XCI influence on disease severity depends on *MECP2* genotype with a correlation between XCI and severity observed only in individuals with *MECP2* variants associated with increased clinical severity. This normalized measure of RTT severity provides the opportunity for future discovery of additional factors contributing to disease severity that may be masked by age and genotype effects.

## 1. Introduction

Rett syndrome (RTT) is a severe neurodevelopmental disorder that predominantly, but not exclusively, affects females and is characterized by apparently normal early development followed by regression of purposeful hand use and acquired verbal communication, development of repetitive hand stereotypies, and impaired gait or the inability to walk [1,2]. With an incidence of 1 in 10,000 live female births, RTT poses a considerable clinical and financial burden on affected individuals and their families, culminating in mean-yearly healthcare costs approaching USD 46,000 per individual [3,4]. Disease burden in RTT is primarily driven by the lack of a cure for the disorder, although approved targeted therapies (e.g., Trofinetide) and symptomatic treatments (e.g., antiepileptics) can improve clinical presentation [5,6,7]. Additional symptoms can be managed through surgical interventions, including spinal fusion to correct scoliosis and gastronomy tube placement to address feeding concerns common among individuals with RTT [8,9]. While these interventions are effective in their own domains, they do not cure nor resolve the molecular basis of the disorder.

Most cases of RTT are caused by de novo heterozygous pathogenic loss-of-function variants in the X-linked transcriptional regulator *Methyl-CpG Binding Protein 2* (*MECP2*) [10,11,12]. Although all people with RTT share these characteristic clinical features, overall clinical severity is variable between affected individuals [13,14]. At the group level, *MECP2* genotype–phenotype relationships have been shown to be a major driver of this clinical variability, with some *MECP2* variants (e.g., R133C, R294X, and R306C) associated with milder clinical severity and other variants (e.g., R106W, T158M, R168X, R255X, and R270X) associated with increased clinical severity [13,15,16]. Although this genotype–phenotype relationship is robust at the group level, some affected individuals display clinical severity discordant from expected severity predicted by this group-level genotype–phenotype relationship [13,15,16,17]; for example, some people with “severe” *MECP2* variants have mild clinical severity and vice versa. This individual-level variation suggests that additional factors such as differences in environment, treatment interventions, or biological/genetic features play a role in determining individual-level clinical severity in RTT. 

Because most cases of RTT are caused by de novo heterozygous variants in the X-linked gene *MECP2*, differential X chromosome inactivation (XCI) has been proposed as a source of variation in clinical severity. During female embryonic development, XCI randomly silences one X chromosome in each cell [18]. In RTT, this results in cellular mosaicism, with some cells expressing a wild-type copy of *MECP2* and others expressing a disease-causing *MECP2* allele [19]. In the majority of RTT cases, de novo pathogenic *MECP2* variants arise on the paternal X chromosome during spermatogenesis, and increased silencing of the diseased paternal allele (pXCI) is expected to result in milder disease severity owing to more cells expressing functional MeCP2 [20,21,22]. In extreme cases, rare incidences of familial RTT have been reported where an unaffected mother has a disease-causing variant in *MECP2* but is protected by preferential inactivation of the mutant allele [11,23,24]. 

The impact of pXCI on disease severity in the general RTT population is less clear. One study restricted analysis to affected individuals with either T158M or R168X variants and found that increased activation of the X chromosome harboring the disease allele correlates with increased severity [25]. However, a larger study including multiple *MECP2* variants did not find a correlation between pXCI and clinical severity [26]. Additional studies reported conflicting results of the effect of non-random XCI on disease severity, although these studies were limited by the lack of information of the directionality of XCI skewing related to mutant *MECP2* allele expression [27,28]. Furthermore, previous studies also had limitations related to the fact that measures of clinical severity in RTT are correlated with both age and specific *MECP2* variants, with a clear age-dependent increase in clinical severity and well-established genotype–phenotype relationships that may outweigh the contribution of pXCI [13,15]. Ideally, evaluation of the effect of XCI on clinical severity in RTT would use age- and genotype-matched cohorts; however, in a rare disorder such as RTT, with a limited population this is not feasible. While previous work addressed the genotype–phenotype issue by restricting analysis to the recurrent T158M and R168X variants, variation in genotype frequency limits the feasibility of this approach for other variants [25,29].

To circumvent the problem of age- and genotype-dependent effects on severity, we utilized data from the US Rett Syndrome Natural History Study (RNHS) to develop age- and genotype-normalized clinical severity scores. Applying this normalized clinical severity score, we found that pXCI had a minor influence on severity in individuals with severe *MECP2* variants but did not contribute to clinical severity in individuals with mild *MECP2* variants. The ability to utilize this normalized clinical severity score provides the opportunity to compare severity across ages and *MECP2* genotypes, and has the potential to help identify other biological or environmental factors that contribute to overall clinical severity in RTT in future studies.

## 2. Materials and Methods

### 2.1. Participants

Participants in this study were enrolled in the Rett syndrome and RTT-related Disorders Natural History Study (RNHS, NCT00299312, NCT02738281), a longitudinal investigation into caregiver-provided historical and clinically observed information spanning from 2006 to 2021. A total of 1826 individuals participated in the RNHS with an average of 5 visits per individual (ranging from 1 to 17 visits). In addition to following individuals with RTT (classic or atypical), this study included people who did not meet RTT diagnostic criteria but had pathogenic variants in *MECP2*, and individuals with RTT-related disorders including *MECP2* duplication syndrome, *CDKL5* deficiency disorder, and *FOXG1* syndrome. 

The Clinical Severity Score (CSS) was employed as one metric to track severity progression in RNHS participants. The CSS is a summation of 13 individual clinical parameters assessed with Likert scores from 0 to 4 or 0 to 5. The total CSS score ranges from 0 to 58, with 0 corresponding to normal and 58 representing the most severe presentation of the disorder [13]. Clinicians rating the CSS were provided in-person training at site visits to ensure consistent scoring across study locations.

### 2.2. Data Used for This Manuscript

Longitudinal CSS and individual parameters used to calculate the CSS were retrieved from the RNHS. A systematic algorithmic method was used to identify and revise longitudinal data on individual CSS entries in the RNHS that were logically inconsistent to generate revised longitudinal CSS scores for 1819 individuals (7 individuals from the total RNHS had missing data, precluding further processing). A schematic of the data correction algorithm is presented in Supplementary Table S1. Briefly, individuals who showed variation in fixed historical scores (e.g., Age of Onset of Regression) were isolated and values deviating from the earliest reported observation were replaced with this value. For metrics which can only increase with time or follow a plateau (e.g., Scoliosis), values that showed a decrease or deviation from the plateau were replaced with the nearest previous observation. Revised CSS scores were calculated from these cleaned parameters for use in downstream analysis.

### 2.3. XCI Data Generation

Paternal X chromosome inactivation status was obtained as part of a previously published study [26]. Briefly, maternal and proband peripheral blood samples were obtained as part of the RNHS (NCT02738281) under the protocol Biobanking of Rett Syndrome and Related Disorders (NCT02705677). DNA samples isolated from peripheral blood samples were analyzed for repeat length and methylation status at the AR locus [30]. The percentage of paternal X inactivation was extrapolated by comparing maternal and proband samples. From 320 individuals with available XCI records in the RNHS, 198 had an informative XCI result and met restrictions for the current study as outlined above.

### 2.4. CSS Model Development

Data processing and analysis were performed using R version 4.1.3. The analysis is readily conducted using the provided R code and supplemental data by someone proficient in using R and associated packages.

#### 2.4.1. Model Development

The statistical analysis training was restricted to female individuals with a confirmed pathogenic variant in *MECP2* belonging to one of the eleven common variant groupings (R106W, R133C, T158M, R168X, R255X, R270X, R294X, R306C, large deletion, early truncation, or C-terminal truncating variant). Participants were also restricted to those with visits between 2 and 25 years of age to account for low numbers of observations beyond this window. A total of 1178 individuals met these requirements for downstream analysis (1003 with a diagnosis of “Classic” and 175 with a diagnosis of “Atypical”; see Table 2 and Supplementary Table S2 for demographic information). Following further restriction to individuals with a classic diagnosis for purposes of model training, 5469 observations across 1003 individuals were available for model development, with an average of 5 observations per individual. 

A mixed effects model was chosen for further analysis to accommodate the longitudinal nature of the data and between-subject differences in observation number and interval of observation [31]. Exploratory data analysis indicated CSS progression follows a logarithmic growth-like pattern (Supplementary Figure S1), so age was log transformed to accommodate this observation. Transformed age values were further decomposed into between- and within-person components to parse out subject versus group effects [32,33,34]. The between-person component was calculated from the centered mean transformed age of observation within an individual (“Mean_lnage_center”, while the within-person component (“Duration”) was defined as the distance from the between-person component to the transformed age of observation within an individual. A conditional growth mixed effects model of *MECP2* genotype and age impact on clinical severity (1) was fitted to the transformed decomposed data using the lme function within the nlme package. In this model, age was defined as the interaction of between- and within-subject time components, with only the within-subject component considered for subject-specific random effects. Mean genotype-specific clinical severity trajectories were calculated using the predict function from the nlme package to provide a top-level view of CSS progression with time for each common *MECP2* genotype.

 Level 1  *Y_ij_* = *β*_0*j*_ + *β*_1*j*_*t_ij_* + *R_ij_*                            *t_ij_* = *Mean*_*lnage*_*center_j_* + *Mean*_*lnage*_*center_j_* : *Duration_ij_* + *Duration_ij_*        Level 2  *β*_0*j*_ = *γ*_00_ + *γ*_01_*MECP2*_*genotype_j_* + *U*_0*j*_              *β*_1*j*_ = *γ*_10_ + *γ*_11_*MECP2*_*genotype_j_* + *U*_1*j*_ (1)

#### 2.4.2. Model Validation

A pseudo leave-one-out cross-validation approach was used to determine model accuracy. Model performance was assessed on any female with one of the above variants regardless of diagnosis (1178 individuals total). The model was iteratively trained on individuals with a classic diagnosis, leaving out the test subject if they had a classic diagnosis. For novel subjects with an atypical diagnosis, no exclusion was warranted. A total of 1178 test subject–model pairs were run. Individual level predictions were made for each test subject–model pair using the IndvPred_lme function in the JMbayes package to allow Bayesian prediction of the novel test subject [35]. The root mean squared error was calculated by standard methods for predicted and observed values for each individual. For a subset of individuals with numerous observations, individual-level predicted trajectories were plotted alongside observed CSS values for visual inspection (Supplementary Figure S2).

#### 2.4.3. CSS Trajectory Prediction

An overarching model was trained on individuals with a classic diagnosis as described in Model Development. Using the IndvPred_lme function in the JMbayes package, predicted clinical severity scores were calculated for ages 2 to 25 in 0.1-year bins for all 1178 individuals in this study regardless of diagnosis. Mean predicted CSS values were then calculated for each individual by averaging predicted severity scores across time. The mean predicted CSS is analogous to calculating the area under the curve of a predicted severity trajectory over time, as equal numbers of predicted scores are calculated for each individual over the same intervals. The mean predicted CSS therefore provides an integrated view of severity progression without retaining age as a factor, effectively normalizing the score across ages. 

#### 2.4.4. CSS Percentile Estimation and Normative CSS Calculation

Mean predicted CSS scores for individuals with a classic diagnosis were isolated and cumulative distributions of mean predicted CSS were calculated for each variant group using the ecdf function in the stats package. Using these genotype specific cumulative distributions of CSS, percentiles were assigned to the mean predicted CSS scores for all individuals in this study regardless of diagnosis. This mean percentile score was defined as normative CSS (nCSS), an age- and genotype-normalized clinical severity score, in downstream analysis.

### 2.5. Analysis of XCI Effect

Pearson’s correlation coefficients were calculated separately for the association of paternal XCI with CSS for all individuals, those with mild *MECP2* variants, and those with severe variants. Potential interactions between *MECP2* variant severity and XCI were assessed by regression analysis of a simplified model of normative CSS:

*nCSS* = *SevGroup* + *SevGroup* : *pXCI* + *pXCI* (2)

where *nCSS* is the age- and genotype-invariant normative severity score described above, *SevGroup* is the *MECP2* variant severity grouping (mild or severe), and *pXCI* is the percentage of paternal X inactivation. Percentage variance explained by the simplified model was determined by dividing an individual parameter’s sum of squares by the model total sum of squares.

### 2.6. Data and Code Availability

Revised CSS scores, model predicted nCSS scores, and pXCI (if available) are provided in the Supplementary Materials. Code employed for model development, analysis, and figure generation is available at https://github.com/jkmerrit/2023_RTT_XCI (accessed on 19 March 2024).

## 3. Results

### 3.1. pXCI Does Not Correlate with Raw CSS Scores

To evaluate the effect of skewed X chromosome inactivation (XCI) on severity in RTT, we obtained XCI information on 320 people with RTT and maternal XCI data, which allows the determination of paternal XCI (pXCI) in the affected individuals. For the 320 affected probands, informative pXCI results were obtained for 198 participants with RTT (Table 1, Classic RTT *n* = 183, Atypical *n* = 15). 

**Figure 1:**
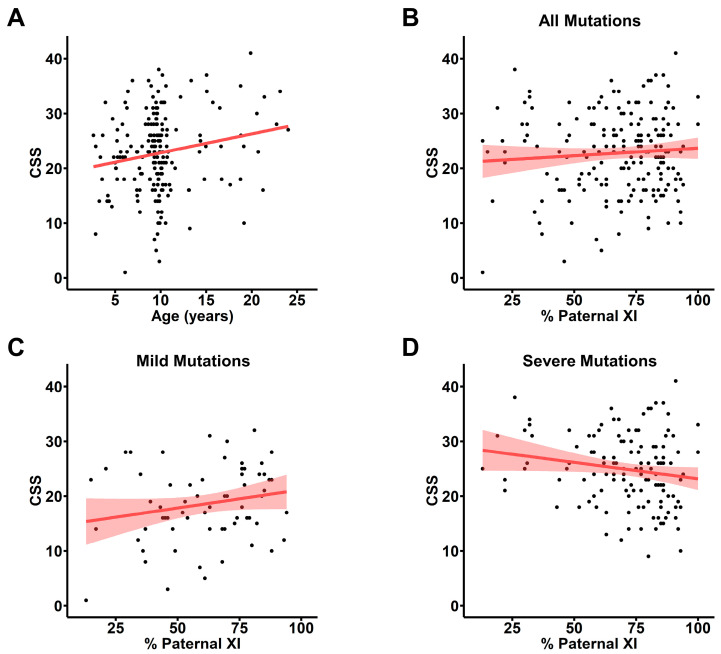
X chromosome inactivation does not correlate with raw clinical severity scores. (**A**) Raw clinical severity scores (CSS) at visit closest to 9.7 years old, the mean age of all visits for the XCI cohort. Linear regression (red line) shows an age-dependent increase in severity. (**B**) Raw CSS scores plotted against percentage of pXCI for all participants (*n* = 198, red line: linear regression of plotted values, shaded area: standard error) showing no correlation (Pearson’s r = 0.074; *p =* 0.298; 95% CI: −0.066 to 0.212). (**C**) No correlation between pXCI and raw CSS for individuals with mild *MECP2* variants (R133C, R294X, R306C, or CTT; *n* = 66; Pearson’s r = 0.20; *p =* 0.103; 95% CI: −0.042 to 0.424). (**D**) A trend towards a weak negative correlation between pXCI and raw CSS is seen for individuals with severe *MECP2* variants (early truncation, R106W, T158M, R168X, R255X, R270X, or large deletions; *n* = 132; Pearson’s r = −0.17; *p =* 0.055; 95% CI: −0.329 to 3.33 × 10^−3^).

To account for the observed age-dependent increase in clinical severity in the entire cohort of people with RTT enrolled in the RNHS (Figure S1A), we assessed the association between pXCI and CSS score observed nearest to 9.7 years of age, the mean all-visit age for the cohort with informative pXCI information. For many participants with informative pXCI, the nearest CSS score was several years removed from the 9.7-year-old mean, and we observed the same overall increase in CSS with age in this group (Figure 1A). Using the CSS score closest to 9.7 years old for participants with informative pXCI data did not show any correlation between pXCI and CSS (Figure 1B). To control for known *MECP2* genotype–phenotype relationships where some variants have a mild phenotype (R133C, R294X, R306C, C-terminal truncations; Supplementary Figure S1B,C) and others present a more severe disorder (R106W, T158M, R168X, R255X, R270X, early truncations, and large deletions), we looked at pXCI/CSS relationships separately within these groups. While no correlation is present in individuals with mild *MECP2* variants (Figure 1C), a trend towards a negative correlation exists in individuals with severe variants (Figure 1D). The presence of age and genotype effects in this analysis may mask less salient contributors to RTT severity and obscure true pXCI/CSS relationships, highlighting the necessity of a severity metric independent of these factors.

### 3.2. Modeling Accurately Predicts Rett Syndrome Severity over Time

**Figure 2:**
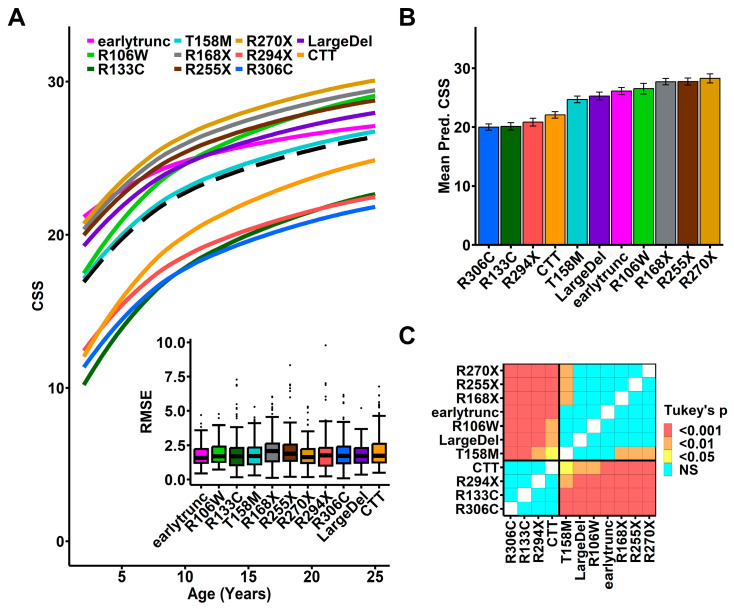
Mixed effects model of Rett Syndrome clinical severity. (**A**) Best-fit model of age-related CSS trajectory for all participants (black dotted line) and individuals with common variants (colored lines for variant groups as shown in legend). Inset: Root mean squared error (RMSE) for mixed effects model-predicted clinical severity scores determined by leave-one-out cross-validation. Bars: median, minimum, and maximum RMSE, boxes: interquartile range, dots: outliers (**B**) Within-subject mean predicted CSS (+/− SEM) for variant groups (*X*-axis) recapitulates previously established differences in severity between *MECP2* variant groups. (**C**) Heatmap showing pairwise comparisons of mean predicted CSS by variant (ANOVA with post hoc pairwise comparisons using Tukey’s HSD), with color indicating *p*-value cutoffs as indicated in panel legend.

To develop an age- and genotype-normalized CSS, we constructed a mixed effects model incorporating the contribution of assessment age and *MECP2* genotype on CSS score, which enables the prediction of an individual’s CSS at any age from a limited set of observations. To generate this model, we focused on individuals in the RNHS with classic RTT and common *MECP2* variants or variant groups to model the typical clinical severity progression (*n* = 1003; Table 2 red dashed area; Table S2). Because the observed age-dependent increase in CSS follows a logarithmic growth pattern (Figure S1A), we incorporated a logarithmic transform for time. To accommodate variation in observed severity trajectories between individuals, we incorporated individual identifiers as a random effect, and decomposed time in the model into two components: the mean age of observation (between-subject effects) and the length of time separating an individual observation from the mean age (within-subject effects) [33]. Acknowledging known *MECP2* genotype–phenotype relationships, we treated genotype as a fixed effect in the model such that each variant grouping would have a separate baseline intercept and trajectory consistent with clinical observations [13,15]. This accurately modeled the expected rapid increase in severity from 2 to 10 years of age with subsequent deceleration in the rate of CSS increase and approaching a plateau in later years (Figure 2A, black dashed line overall group). For individual variants, the model produced similar growth trajectories with a pattern of genotype-specific severity consistent with previous reports (Figure 2A, colored lines) [13,15].

To validate the model, we assessed the accuracy of individual participant model predicted scores compared to observed CSS scores. Visual inspection of predicted individual participant-level severity trajectories showed a close relationship to observed CSS scores for all common variant groups (example images shown in Figure S2). We empirically assessed the accuracy of this model using a leave-one-out cross-validation approach to determine the predictive error (root mean square error [RMSE]) of the model for all individuals with RTT (classic and atypical) in the RNHS (*n* = 1178, Table 2 and Table S1). The overall RMSE was ~2, indicating good fit and demonstrating that the model is able to predict an individual’s CSS within ± 2 points on the 58-point CSS (Figure 2A inset). Using individual mean predicted CSS as an age-normalized score, we found *MECP2* genotype–phenotype relationships seen with the observed CSS score (Supplementary Figure S1B,C) were preserved using the individual model-predicated mean CSS score (Figure 2B,C), demonstrating that the model accurately represents observed RTT severity. However, this genotype–phenotype relationship highlights the need for a genotype-normalized measure of RTT severity to enable comparison across genotypes as well as ages.

### 3.3. Age- and Genotype-Normalized CSS Scores Reveal a Genotype-Dependent Correlation between pXCI and Severity

**Figure 3:**
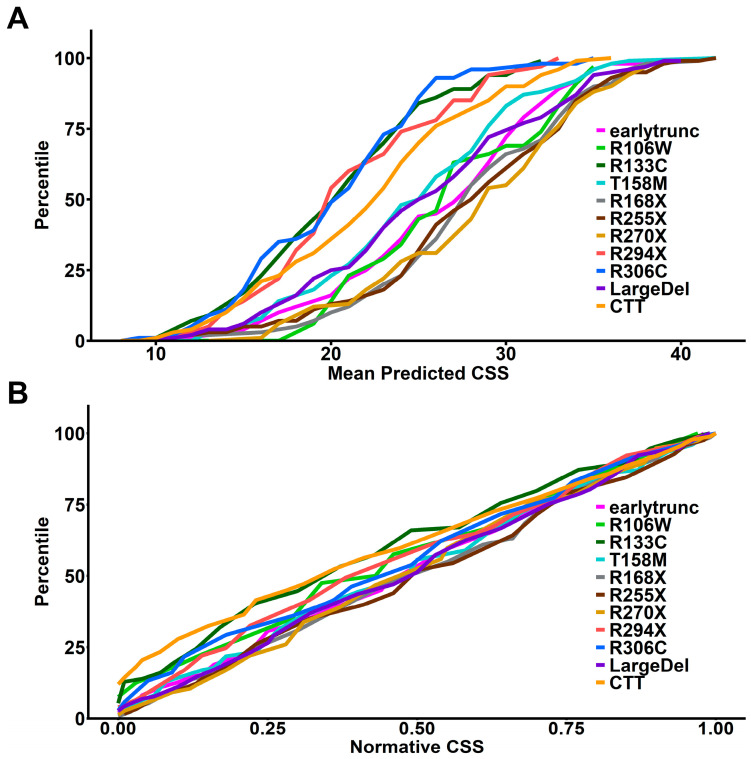
Generation of variant-specific normative CSS scores. (**A**) Cumulative distributions of within-subject mean predicted CSS for MECP2 variant groups showing expected genotype–phenotype relationship as presented in Figure 2. Normative CSS for each participant is generated by determining the variant group-specific percentile for the participant’s mean predicted CSS. (**B**) Cumulative distributions of normative CSS scores by MECP2 variant group demonstrates expected loss of genotype–phenotype relationship for variant-specific normative CSS scores.

To control for *MECP2* genotype effects on disease severity, we created a genotype-normalized CSS by calculating the within-genotype cumulative distribution of age-normalized mean predicted CSS scores from participants with classic RTT and common *MECP2* variants or variant groups (Figure 3A). This allows the conversion of the age-normalized mean predicted CSS score to a standardized *MECP2* variant-specific score based on the percentile within the variant-specific cumulative distribution (percentile/100, thus ranging from 0 to 1). We then calculated the *MECP2* genotype-normalized scores for all RTT participants (classic and atypical) from individual model-predicted mean CSS scores. Using these genotype-normalized scores as a normative CSS (nCSS) removes the genotype–phenotype impact on severity, as demonstrated by the cumulative distribution plot of nCSS (Figure 3B), which lacks the genotype–phenotype relationship observed in Figure 3A. This nCSS (which incorporates both age and genotype effects) allows for standardized comparison of clinical severity in RTT between individuals at different ages and with different *MECP2* genotypes.

**Figure 4:**
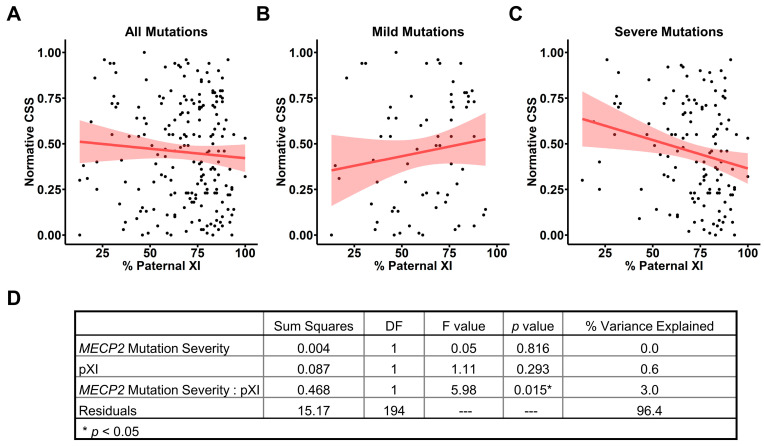
Paternal X inactivation correlates with reduced normative CSS for severe *MECP2* variants. (**A**) Normative CSS plotted against pXCI for all individuals shows no correlation (*n* = 198; Pearson’s r = −0.073; *p* = 0.310; 95% CI: −0.210 to 0.068). Regression line and standard error shading as in Figure 1. (**B**) pXCI shows no correlation with normative CSS scores in individuals with mild *MECP2* variants (*n* = 66; Pearson’s r = 0.139; *p =* 0.265; 95% CI: −0.106 to 0.369). (**C**) Increased pXCI shows a minor correlation with reduced clinical severity as measured by normative CSS in individuals with severe *MECP2* variants (*n* = 132; Pearson’s r = −0.215; *p* = 0.013; 95% CI: −0.372 to −0.0456). (**D**) Summary of a simplified model where normative CSS is described by the interaction between pXCI and *MECP2* variant severity.

We then used this age- and genotype-normalized nCSS to evaluate the contribution of pXCI to clinical severity in RTT. For the entire cohort with informative pXCI data (Table 1), we did not find a relationship between pXCI and nCSS (Figure 4A), consistent with the lack of an observed relationship between raw CSS scores and pXCI (Figure 1A). Similarly, we found no relationship between pXCI and nCSS scores in individuals with mild *MECP2* variants (Figure 4B), as observed for raw CSS and pXCI (Figure 1C). However, we found that pXCI was negatively correlated with nCSS scores for people with severe variants in *MECP2* (Figure 4C, *p* = 0.013, r = −0.215), whereas there was only a trend towards the correlation between raw CSS and pXCI in this group (Figure 1C, *p* = 0.055, r = −0.17). The presence of a unique pXCI/nCSS relationship in the context of severe *MECP2* variants (Figure 4C) suggests pXCI impacts disease severity through an interaction with *MECP2* variant severity. To further explore this relationship, we assessed how pXCI and *MECP2* variant severity contribute to the nCSS score (Figure 4D). As expected, *MECP2* variant severity alone does not affect normative severity due to elimination of genotype–phenotype effects in the nCSS (see Figure 3B). pXCI alone was also not found to significantly explain variation in nCSS between individuals (Figure 4D), consistent with the lack of correlation between pXCI/nCSS in the entire cohort (Figure 4A). However, we found the interaction between *MECP2* variant severity and pXCI notably contributes to nCSS and explains ~3% of the variation in severity between individuals (Figure 4D).

## 4. Discussion

Here we describe a model of RTT severity that allows for generation of an age- and genotype-normative CSS. We show that application of this method to analysis of an XCI dataset reveals a previously unidentified correlation between increased pXCI and decreased disease severity in individuals with severe *MECP2* variants, but not those with mild variants. From the current data, we estimate that three percent of the variation in severity between individuals with RTT is attributed to the interaction between pXCI and *MECP2* variant severity driven by the specific interaction of pXCI and severe variants.

While previous analysis of the same XCI dataset did not identify a correlation between XCI and disease severity, our ability to uncover a relationship in this study is aided by the use of an age- and genotype-normalized severity score, in addition to restricting our analysis to common *MECP2* variants [26]. Our findings are consistent with previous reports of a correlation between XCI and severity in individuals with R168X and T158M, variants that are classified as severe in the current study [25]. Although a higher degree of correlation between XCI and disease severity was previously reported for these variants, the current study encompasses variants beyond R168X and T158M and employs a different severity scale, so only qualitative comparisons between these two works can be made. 

A key limitation in this and previous investigations into the contribution of XCI to RTT severity is the use of peripheral blood as the source of genomic DNA for determining XCI status. Concerns have been raised that XCI in peripheral blood may not accurately represent states of mosaicism in the central nervous system relevant to RTT [27,36,37]. However, recent analysis by the GTEx consortium suggests XCI trends in peripheral blood are maintained in the brain [38]. Having evidence that peripheral blood samples accurately reflect CNS mosaicism supports continued use of whole blood as a surrogate for XCI in the brain, as obtaining suitable numbers of CNS samples for a well-powered investigation of the impact of pXCI on RTT severity is not feasible.

The interaction between *MECP2* variant severity and XCI contributing to nCSS is an unexpected finding. Genotype–phenotype relationships are collapsed in the nCSS (Figure 3B), so the presence of a genotypic interaction with XCI suggests a more complex association. Mild variants invariant to the effects of XCI might represent a base level of *MECP2* dysfunction sufficient to cause RTT. The presence of even relatively small numbers of mild mutant allele-expressing cells elicits hallmarks of the disorder; however, disease severity is largely unaffected by the relative ratio of cells expressing healthy versus mild mutant alleles. Conversely, for more severe variants, a greater ratio of dysfunctional cells could have an additive effect on worsening disease severity. Preferential skewing of XCI and silencing of the disease allele in this case provides a measurable reduction in clinical severity. This is a key consideration as we enter the era of *MECP2*-targeted therapeutics ranging from gene therapy to X chromosome reactivation [39,40,41,42,43]. Our findings suggest *MECP2* variant-specific differences in the percentage of functional *MECP2*-expressing cells needed to elicit observable clinical improvement. For people with mild variants, expression of functional *MECP2* in a large percentage of cells may be required for observable clinical improvement, whereas individuals with severe variants may see clinically meaningful differences with a smaller percentage of functional *MECP2*-expressing cells. 

Beyond exploring impacts of XCI on disease severity, the age- and genotype-normalized nCSS score provides a means to assess other factors that contribute to RTT severity. Because the nCSS score allows for direct comparisons between individuals regardless of their age and *MECP2* variant, we are able to conduct well-powered studies without restriction to specific ages and/or genotypes. This feature also allows for analysis of historical datasets where sample collection may not have been age matched or controlled for genotype. Moreover, the nCSS allows for interrogation of factors contributing to disease severity that may have a relatively small impact on clinical presentation compared to the well-established *MECP2* genotype–phenotype relationship. This approach could contribute to the identification of other biological factors (genetic, metabolomic), environmental factors, or therapies (physical, occupational, speech) that influence clinical severity, but may be masked by the strong *MECP2* genotype–phenotype relationship. Finally, our strategy of modeling longitudinal data to obtain age- and genotype-normalized scores could see application to other measures of clinical outcomes in RTT, including the Revised Motor Behavior Assessment (RMBA), the Rett Syndrome Behavior Questionnaire (RSBQ), and the Rett Syndrome Caregiver Assessment of Symptom Severity (RCASS) [44,45,46].

## Figures and Tables

**Table 1:** X chromosome inactivation study cohort. Phased XCI records were available for 320 individuals with RTT and related disorders. A total of 198 individuals met the restriction of having one of eleven common RTT-causing MECP2 variants. CTT: C-terminal Truncation.

	Participants	Classic RTT	Atypical RTT	Age of First Visit (Years, Mean ± SD)
Early Truncation	17	16	1	5.7 ± 6.2
R106W	8	7	1	6.7 ± 4.5
R133C	10	9	1	6.1 ± 5.6
T158M	26	26	0	7.8 ± 6.5
R168X	25	25	0	5.9 ± 4.3
R255X	24	23	1	5.7 ± 5.3
R270X	14	13	1	6.0 ± 4.3
R294X	16	14	2	8.2 ± 4.2
R306C	19	17	2	7.2 ± 4.8
Large Deletion	18	17	1	6.9 ± 6.4
CTT	21	16	5	6.6 ± 5.4
Total	198	183	15	6.6 ± 5.3

**Table 2:** Model development and validation cohort. RNHS participants were restricted to female individuals with one of eleven common *MECP2* variants and at least one visit between the ages of 2 and 25. These individuals were further restricted to those with a diagnosis of classic RTT for model training (red box).

	Participants	Classic RTT	Atypical RTT	Age of First Visit (Years, Mean ± SD)	Study Duration (Years, Mean ± SD)
Early Truncation	111	100	11	7.6 ± 6.1	5.2 ± 3.9
R106W	40	35	5	5.5 ± 4.2	5.8 ± 4.4
R133C	94	70	24	8.2 ± 6.1	4.3 ± 4.2
T158M	124	119	5	8.8 ± 6.4	5.1 ± 3.9
R168X	133	122	11	7.3 ± 6.2	4.7 ± 4.3
R255X	123	111	12	6.9 ± 5.3	5.1 ± 4.1
R270X	77	67	10	7.3 ± 5.4	4.4 ± 3.8
R294X	77	65	12	10.4 ± 6.0	4.9 ± 4.4
R306C	106	92	14	8.0 ± 5.7	5.1 ± 4.6
Large Deletion	117	102	15	8.0 ± 6.0	4.8 ± 3.9
CTT	176	120	56	8.9 ± 5.9	4.2 ± 3.8
Total	1178	1003	175	8.0 ± 5.9	4.8 ± 4.1

## Data Availability

Revised CSS scores, model predicted nCSS scores, and pXCI (if available) are provided in Supplementary File S2. Code employed for model development, analysis, and figure generation is available at https://github.com/jkmerrit/2023_RTT_XCI (accessed on 19 March 2024).

## Supplementary Materials

The following supporting information can be downloaded at: https://www.mdpi.com/article/10.3390/genes15050594/s1, Figure S1: Rett syndrome severity increases with age and depends on *MECP2* genotype; Figure S2: Example model-predicted clinical severity trajectories for individuals with repeated measures; Table S1: Summary of data corrections used in this study; Table S2: Study participant demographics.

## References

[1] Neul J.L., Kaufmann W.E., Glaze D.G., Christodoulou J., Clarke A.J., Bahi-Buisson N., Leonard H., Bailey M.E.S., Schanen N.C., Zappella M. (2010). Rett Syndrome: Revised Diagnostic Criteria and Nomenclature. Ann. Neurol..

[2] Rett A. (2016). On a Remarkable Syndrome of Cerebral Atrophy Associated with Hyperammonaemia in Childhood. Wien. Med. Wochenschr..

[3] May D., Kponee-Shovein K., Mahendran M., Downes N., Sheng K., Lefebvre P., Cheng W.Y. (2023). Epidemiology and Patient Journey of Rett Syndrome in the United States: A Real-World Evidence Study. BMC Neurol..

[4] Petriti U., Dudman D.C., Scosyrev E., Lopez-Leon S. (2023). Global Prevalence of Rett Syndrome: Systematic Review and Meta-Analysis. Syst. Rev..

[5] Abbas A., Fayoud A.M., El Din Moawad M.H., Hamad A.A., Hamouda H., Fouad E.A. (2024). Safety and Efficacy of Trofinetide in Rett Syndrome: A Systematic Review and Meta-Analysis of Randomized Controlled Trials. BMC Pediatr..

[6] Neul J.L., Percy A.K., Benke T.A., Berry-Kravis E.M., Glaze D.G., Marsh E.D., Lin T., Stankovic S., Bishop K.M., Youakim J.M. (2023). Trofinetide for the Treatment of Rett Syndrome: A Randomized Phase 3 Study. Nat. Med..

[7] Vignoli A., Savini M.N., Nowbut M.S., Peron A., Turner K., Briola F.L., Canevini M.P. (2017). Effectiveness and Tolerability of Antiepileptic Drugs in 104 Girls with Rett Syndrome. Epilepsy Behav..

[8] Menachem S., Hershkovich O., Ackshota N., Friedlander A., Givon U., Ben-Zeev B., Caspi I. (2023). Scoliosis in RETT Syndrome: A National Referral Centre Experience. Clin. Spine Surg..

[9] Downs J., Wong K., Ravikumara M., Ellaway C., Elliott E.J., Christodoulou J., Jacoby P., Leonard H. (2014). Experience of Gastrostomy Using a Quality Care Framework: The Example of Rett Syndrome. Medicine.

[10] Amir R.E., Van den Veyver I.B., Wan M., Tran C.Q., Francke U., Zoghbi H.Y. (1999). Rett Syndrome Is Caused by Mutations in X-Linked MECP2, Encoding Methyl-CpG-Binding Protein 2. Nat. Genet..

[11] Wan M., Lee S.S.J., Zhang X., Houwink-Manville I., Song H.-R., Amir R.E., Budden S., Naidu S., Pereira J.L.P., Lo I.F.M. (1999). Rett Syndrome and Beyond: Recurrent Spontaneous and Familial MECP2 Mutations at CpG Hotspots. Am. J. Hum. Genet..

[12] Bienvenu T., Carrié A., de Roux N., Vinet M.-C., Jonveaux P., Couvert P., Villard L., Arzimanoglou A., Beldjord C., Fontes M. (2000). MECP2 Mutations Account for Most Cases of Typical Forms of Rett Syndrome. Hum. Mol. Genet..

[13] Neul J.L., Fang P., Barrish J., Lane J., Caeg E.B., Smith E.O., Zoghbi H., Percy A., Glaze D.G. (2008). Specific Mutations in Methyl-CpG-Binding Protein 2 Confer Different Severity in Rett Syndrome. Neurology.

[14] Naidu S., Bibat G., Kratz L., Kelley R.I., Pevsner J., Hoffman E., Cuffari C., Rohde C., Blue M.E., Johnston M.V. (2003). Clinical Variability in Rett Syndrome. J. Child Neurol..

[15] Cuddapah V.A., Pillai R.B., Shekar K.V., Lane J.B., Motil K.J., Skinner S.A., Tarquinio D.C., Glaze D.G., McGwin G., Kaufmann W.E. (2014). Methyl-CpG-Binding Protein 2 (MECP2) Mutation Type Is Associated with Disease Severity in Rett Syndrome. J. Med. Genet..

[16] Frullanti E., Papa F.T., Grillo E., Clarke A., Ben-Zeev B., Pineda M., Bahi-Buisson N., Bienvenu T., Armstrong J., Roche Martinez A. (2019). Analysis of the Phenotypes in the Rett Networked Database. Int. J. Genom..

[17] Romano A., Lotan M., Fabio R.A. (2023). A Severity Comparison between Italian and Israeli Rett Syndrome Cohorts. Diagnostics.

[18] Disteche C.M., Berletch J.B. (2015). X-Chromosome Inactivation and Escape. J. Genet..

[19] Van den Veyver I.B., Zoghbi H.Y. (2001). Mutations in the Gene Encoding Methyl-CpG-Binding Protein 2 Cause Rett Syndrome. Brain Dev..

[20] Trappe R., Laccone F., Cobilanschi J., Meins M., Huppke P., Hanefeld F., Engel W. (2001). MECP2 Mutations in Sporadic Cases of Rett Syndrome Are Almost Exclusively of Paternal Origin. Am. J. Hum. Genet..

[21] Chae J.H., Hwang H., Hwang Y.S., Cheong H.J., Kim K.J. (2004). Influence of MECP2 Gene Mutation and X-Chromosome Inactivation on the Rett Syndrome Phenotype. J. Child Neurol..

[22] Miltenberger-Miltenyi G., Laccone F. (2003). Mutations and Polymorphisms in the Human Methyl CpG-Binding Protein MECP2. Hum. Mutat..

[23] Ravn K., Roende G., Duno M., Fuglsang K., Eiklid K.L., Tümer Z., Nielsen J.B., Skjeldal O.H. (2011). Two New Rett Syndrome Families and Review of the Literature: Expanding the Knowledge of MECP2 Frameshift Mutations. Orphanet J. Rare Dis..

[24] Zhang Q., Zhao Y., Bao X., Luo J., Zhang X., Li J., Wei L., Wu X. (2017). Familial Cases and Male Cases with MECP2 Mutations. Am. J. Med. Genet. B Neuropsychiatr. Genet..

[25] Archer H., Evans J., Leonard H., Colvin L., Ravine D., Christodoulou J., Williamson S., Charman T., Bailey M.E.S., Sampson J. (2007). Correlation between Clinical Severity in Patients with Rett Syndrome with a p.R168X or p.T158M MECP2 Mutation, and the Direction and Degree of Skewing of X-Chromosome Inactivation. J. Med. Genet..

[26] Fang X., Butler K.M., Abidi F., Gass J., Beisang A., Feyma T., Ryther R.C., Standridge S., Heydemann P., Jones M. (2022). Analysis of X-inactivation Status in a Rett Syndrome Natural History Study Cohort. Mol. Genet. Genom. Med..

[27] Xiol C., Vidal S., Pascual-Alonso A., Blasco L., Brandi N., Pacheco P., Gerotina E., O’Callaghan M., Pineda M., Armstrong J. (2019). X Chromosome Inactivation Does Not Necessarily Determine the Severity of the Phenotype in Rett Syndrome Patients. Sci. Rep..

[28] Amir R.E., Van Den Veyver I.B., Schultz R., Malicki D.M., Tran C.Q., Dahle E.J., Philippi A., Timar L., Percy A.K., Motil K.J. (2000). Influence of Mutation Type and X Chromosome Inactivation on Rett Syndrome Phenotypes. Ann. Neurol..

[29] Ehrhart F., Jacobsen A., Rigau M., Bosio M., Kaliyaperumal R., Laros J.F.J., Willighagen E.L., Valencia A., Roos M., Capella-Gutierrez S. (2021). A Catalogue of 863 Rett-Syndrome-Causing MECP2 Mutations and Lessons Learned from Data Integration. Sci. Data.

[30] Pegoraro E., Schimke R.N., Arahata K., Hayashi Y., Stern H., Marks H., Glasberg M.R., Carroll J.E., Taber J.W., Wessel H.B. (1994). Detection of New Paternal Dystrophin Gene Mutations in Isolated Cases of Dystrophinopathy in Females. Am. J. Hum. Genet..

[31] Murphy J.I., Weaver N.E., Hendricks A.E. (2022). Accessible Analysis of Longitudinal Data with Linear Mixed Effects Models. Dis. Model. Mech..

[32] Kraemer H.C., Yesavage J.A., Taylor J.L., Kupfer D. (2000). How Can We Learn About Developmental Processes From Cross-Sectional Studies, or Can We?. Am. J. Psychiatry.

[33] Curran P.J., Bauer D.J. (2011). The Disaggregation of Within-Person and Between-Person Effects in Longitudinal Models of Change. Annu. Rev. Psychol..

[34] Hopwood C.J., Bleidorn W., Wright A.G.C. (2022). Connecting Theory to Methods in Longitudinal Research. Perspect. Psychol. Sci..

[35] Rizopoulos D. (2016). The R Package JMbayes for Fitting Joint Models for Longitudinal and Time-to-Event Data Using MCMC. J. Stat. Softw..

[36] Shahbazian M.D., Sun Y., Zoghbi H.Y. (2002). Balanced X Chromosome Inactivation Patterns in the Rett Syndrome Brain. Am. J. Med. Genet..

[37] Zhu X., Li M., Pan H., Bao X., Zhang J., Wu X. (2010). Analysis of the Parental Origin of De Novo MECP2 Mutations and X Chromosome Inactivation in 24 Sporadic Patients With Rett Syndrome in China. J. Child Neurol..

[38] Tukiainen T., Villani A.-C., Yen A., Rivas M.A., Marshall J.L., Satija R., Aguirre M., Gauthier L., Fleharty M., Kirby A. (2017). Landscape of X Chromosome Inactivation across Human Tissues. Nature.

[39] Sadhu C., Lyons C., Oh J., Jagadeeswaran I., Gray S.J., Sinnett S.E. (2023). The Efficacy of a Human-Ready miniMECP2 Gene Therapy in a Pre-Clinical Model of Rett Syndrome. Genes.

[40] Powers S., Likhite S., Gadalla K.K., Miranda C.J., Huffenberger A.J., Dennys C., Foust K.D., Morales P., Pierson C.R., Rinaldi F. (2023). Novel MECP2 Gene Therapy Is Effective in a Multicenter Study Using Two Mouse Models of Rett Syndrome and Is Safe in Non-Human Primates. Mol. Ther..

[41] Carrette L.L.G., Blum R., Ma W., Kelleher R.J., Lee J.T. (2018). Tsix-Mecp2 Female Mouse Model for Rett Syndrome Reveals That Low-Level MECP2 Expression Extends Life and Improves Neuromotor Function. Proc. Natl. Acad. Sci. USA.

[42] Carrette L.L.G., Wang C.-Y., Wei C., Press W., Ma W., Kelleher R.J., Lee J.T. (2018). A Mixed Modality Approach towards Xi Reactivation for Rett Syndrome and Other X-Linked Disorders. Proc. Natl. Acad. Sci. USA.

[43] Przanowski P., Wasko U., Zheng Z., Yu J., Sherman R., Zhu L.J., McConnell M.J., Tushir-Singh J., Green M.R., Bhatnagar S. (2018). Pharmacological Reactivation of Inactive X-Linked Mecp2 in Cerebral Cortical Neurons of Living Mice. Proc. Natl. Acad. Sci. USA.

[44] Raspa M., Bann C.M., Gwaltney A., Benke T.A., Fu C., Glaze D.G., Haas R., Heydemann P., Jones M., Kaufmann W.E. (2020). A Psychometric Evaluation of the Motor-Behavioral Assessment Scale for Use as an Outcome Measure in Rett Syndrome Clinical Trials. Am. J. Intellect. Dev. Disabil..

[45] Percy A.K., Neul J.L., Benke T.A., Marsh E.D., Glaze D.G. (2023). A Review of the Rett Syndrome Behaviour Questionnaire and Its Utilization in the Assessment of Symptoms Associated with Rett Syndrome. Front. Pediatr..

[46] Raspa M., Gwaltney A., Bann C., von Hehn J., Benke T.A., Marsh E.D., Peters S.U., Ananth A., Percy A.K., Neul J.L. (2024). Psychometric Assessment of the Rett Syndrome Caregiver Assessment of Symptom Severity (RCASS). J. Autism Dev. Disord..

