# Supplementary material for: Normalized Clinical Severity Scores Reveal a Correlation between X Chromosome Inactivation and Disease Severity in Rett Syndrome
Source: Genes (Basel). 2024 May 8;15(5):594. doi: 10.3390/genes15050594 (PMC11120815; doi:10.3390/genes15050594)

Supplementary Tables

Table S1. Summary of data corrections used in this study. Of 13 parameters in the CSS, 6 parameters are purely observational and not amenable to correction of logical inconsistencies. The 7 remaining parameters were corrected for deviations likely resulting from user and/or recording errors. A total of 772 individuals out of 1,819 participants required data correction. An average of ~2 parameters required correction in each of these 772 individuals' records.

CSS Parameter	Scale	Corrective Action	# Individuals with corrected entries (n = 1,819)
Age of onset of regression	0-5	Once a score > 0 reported, that score is maintained for all following visits. Deviations are replaced with the previous observed score.	144
Onset of stereotypies	0-4	Once a score is reported, that score is maintained for all following visits. Deviations are replaced with the previous observed score.	144
Head growth	0-4	Score cannot decrease with time if > 1, if a lower score is observed, it is replaced with the previous observed score.	112
Independent sitting at visit by exam	0-5	Once score other than 5 (never acquired) is reported, the score cannot return to 5. Scores are allowed to fluctuate between 4 (skill lost) and previous reported score, but fluctuation between scores 0-3 is not allowed. Deviations are replaced with the previous observed score.	290
Ambulation at visit by exam	0-5	Once score other than 5 (never acquired) is reported, the score cannot return to 5. Scores are allowed to fluctuate between 4 (skill lost) and previous reported score. Scores are allowed to increase within these constraints. Deviations are replaced with the previous observed score.	329
Hand use	0-4	Once score other than 4 (never acquired) is reported, the score cannot return to 4. Scores are allowed to fluctuate between 3 (skill lost) and previous reported score. Deviations are replaced with the previous observed score.	342
Scoliosis	0-5	Once score of 5 (indicative of surgery) is reported, the score should remain 5 for following visits. Deviations are replaced with a score of 5.	9

Table S2. Study Participant Demographics

Participants (n=1178)		Number (%) Ethnicity Non-Hispanic	Hispanic
Race	White	851 (72.2)	128 (10.9)
	African American/Black	49 (4.2)	3 (0.3)
	Asian	46 (3.9)	2 (0.2)
	Native American	5 (0.4)	5 (0.4)
	More than one race	52 (4.4)	9 (0.8)
	Not reported	4 (0.3)	23 (2)
	Refused	1 (0.1)	0 (0)

Classic RTT			
	Ethnicity		
**Race**	Non-Hispanic	Hispanic	Total
White	722	106	828
African American/Black	44	3	47
Asian	38	2	40
Native American	3	5	8
More than one race	47	8	55
Not reported	3	21	24
Refused	1	0	1
Total	858	145	1003
**Atypical RTT**			
	Non-Hispanic	Hispanic	Total
White	129	22	151
African American/Black	5	0	5
Asian	8	0	8
Native American	2	0	2
More than one race	5	1	6
Not reported	1	2	3
Refused	0	0	0
Total	150	25	175

Figure S1. Rett syndrome severity increases with age and depends on *MECP2* genotype. A) Baseline clinical severity scores (CSS) plotted with individual age at baseline for 1,451 female RNHS participants with RTT and an identified causal mutation in *MECP2*. Red line: fitted log curve of plotted data points. B) Mean baseline severity scores for common Rett-causing mutations. Error bars represent SEM. C) Heatmap showing pairwise comparisons of baseline CSS by mutation (ANOVA with post-hoc pairwise comparisons using Tukey's HSD) with color indicating p-value cutoffs as indicated in panel legend.

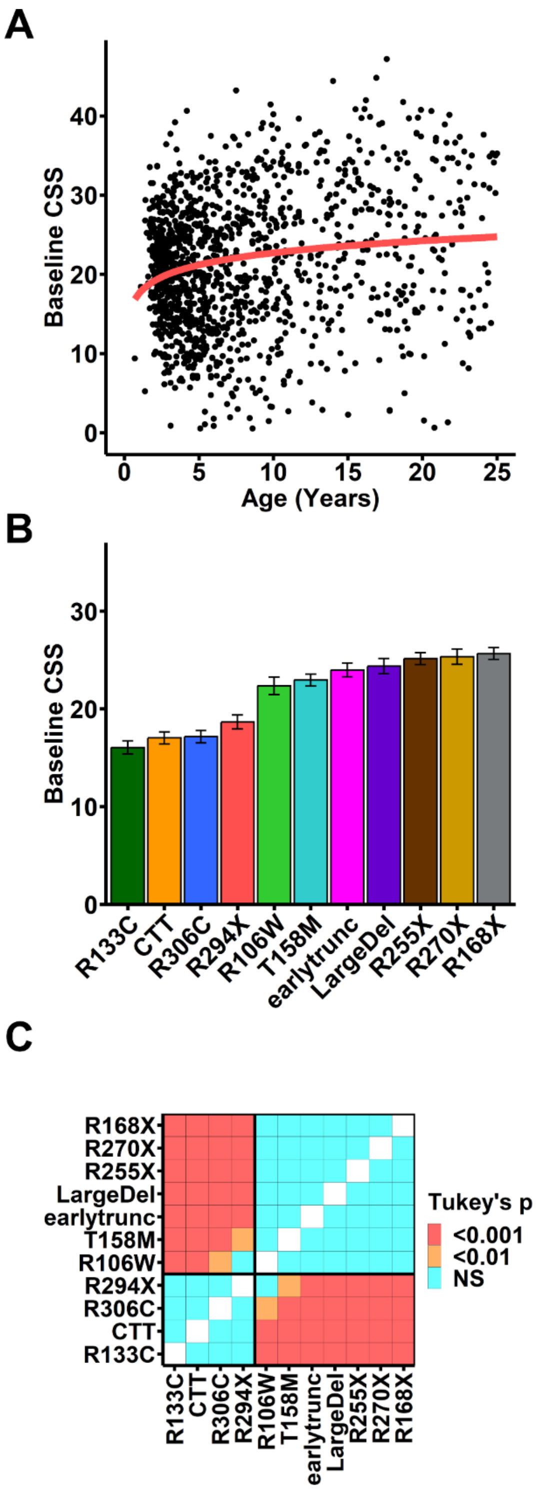

Figure S2. Example model predicted clinical severity trajectories for individuals with repeated measures. Model predicted clinical severity scores (blue line) plotted against observed scores (black points). One individual for each mutation grouping was randomly selected from individuals with greater than thirteen visits in the RNHS to provide a visual assessment of model fit.

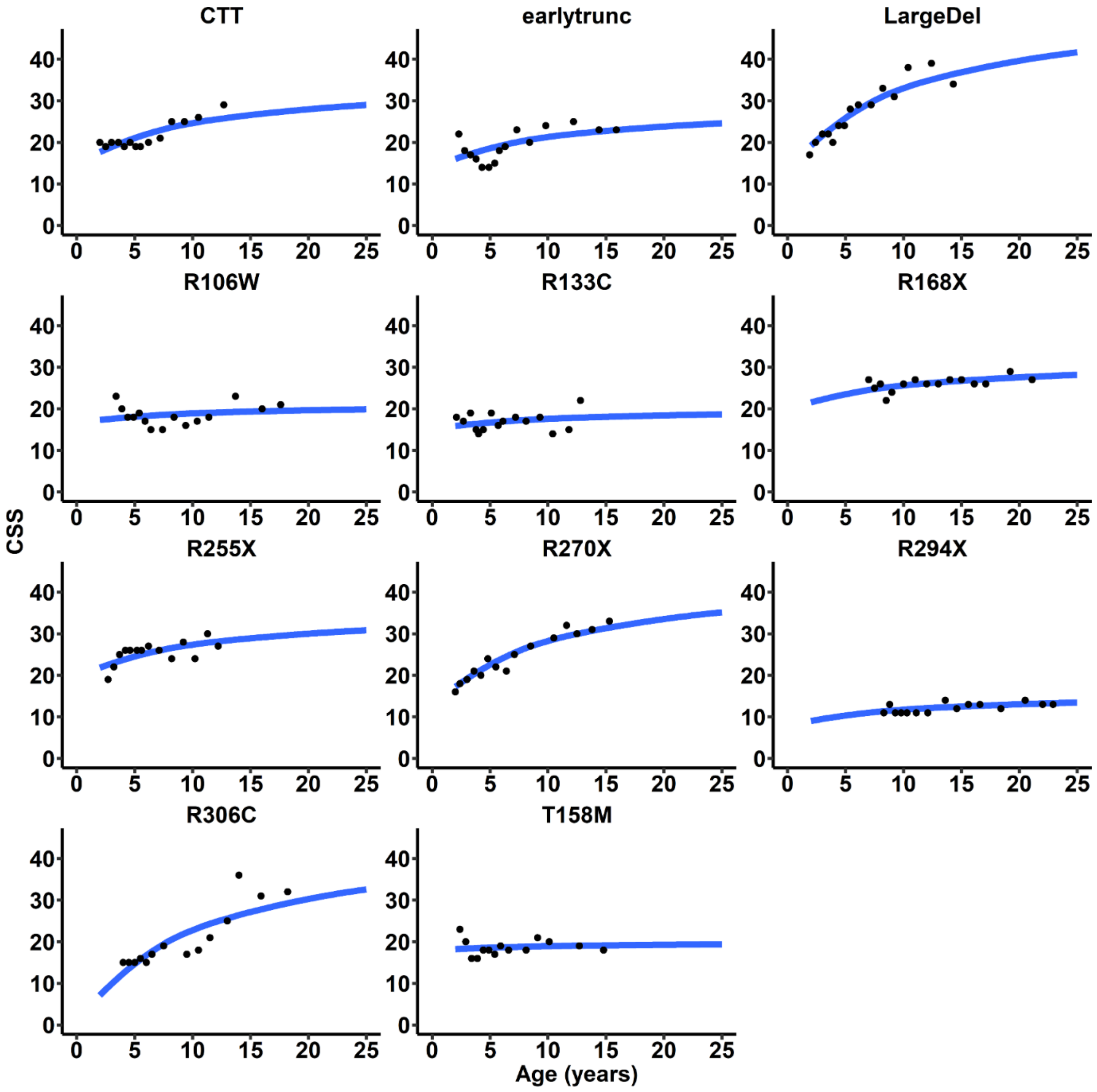

Supplement: Supplementary file 1 [file genes-15-00594-s001.zip › genes-2963669-supplementary.pdf]
